# Supplementary material for: Menstrual hygiene practice among adolescent girls in Ethiopia: A systematic review and meta-analysis
Source: PLoS One. 2022 Jan 4;17(1):e0262295. doi: 10.1371/journal.pone.0262295 (PMC8726503; doi:10.1371/journal.pone.0262295)
Supplement: S2 File — (DOCX) [file pone.0262295.s002.docx]

**Supplementary file 1: Examples of searching strategy**

| **Database** | **Example of searching strategy** |
| --- | --- |
| PubMed | ((((((((((((Adolescent [MeSH Terms]) OR Adolescents [Text Word]) OR adolescent) OR adolescence) OR puberty) OR peer) OR school)) AND (((Menstruation [MesH]) OR menstrual) OR menses))) AND (((((((Hygiene [MeSH]) OR hygiene) OR hygienically) OR sanitation) OR sanitary) OR Feminine Hygiene Products [MesH]) OR Menstrual Hygiene Products [MesH])) AND ((Ethiopia [MeSH Terms]) OR Ethiopia) |
| POPLINE | (Menstrual OR Menstruation OR "menstrual hygiene") AND "Ethiopia" |
| Google Scholar | (“menstrual” OR “hygiene”) AND "Ethiopia" |
| Science Direct | “Menstrual hygiene" AND "Ethiopia” |
| Hinari | (“Menstrual hygiene management” OR “Menstrual hygiene” OR “Menstrual hygiene practice” OR "Menstrual" OR "Hygiene") AND Ethiopia |
| ProQuest | “Menstrual hygiene" AND "Ethiopia” |
| African Journal Online | “Menstrual hygiene" AND "Ethiopia” |
| Direct of Open Access Journals | “Menstrual hygiene" AND "Ethiopia” |
| Cochrane Library | ("Menstrual blood loss [MeSH]" OR "mensural) AND Ethiopia |
