# Supplementary material for: Menstrual hygiene practice among adolescent girls in Ethiopia: A systematic review and meta-analysis
Source: PLoS One. 2022 Jan 4;17(1):e0262295. doi: 10.1371/journal.pone.0262295 (PMC8726503; doi:10.1371/journal.pone.0262295)
Supplement: S3 File — (DOCX) [file pone.0262295.s003.docx]

**Supplementary file 2: Individual quality assessment of 22 articles included in the review of the status of menstrual hygiene management among adolescent girls in Ethiopia, studies published between 2004 and 2021.**

| **Authors, Year** | **Sampling frame** | **Sampling strategy** | **Sample size** | **Description of research setting & population** | **Data analysis conducted with sufficient coverage** | **Valid methods used for the identification of the condition** | **Reliability of the instrument used** | **Statistical analysis methods** | **Response rate** | **Total** | **Risk of bias** |
| --- | --- | --- | --- | --- | --- | --- | --- | --- | --- | --- | --- |
| Birhane AD et al., 2020 | 0 | 0 | 0 | 0 | 0 | 1 | 1 | 0 | 0 | 2 | Low |
| Felleke AA et al., 2021 | 1 | 0 | 0 | 0 | 0 | 1 | 1 | 1 | 0 | 4 | Moderate |
| Kitesa B et al., 2016 | 0 | 0 | 0 | 0 | 0 | 1 | 1 | 0 | 0 | 2 | Low |
| Zeleke B, 2016 | 0 | 0 | 0 | 0 | 0 | 1 | 1 | 0 | 0 | 2 | Low |
| Biruk E, 2017 | 0 | 0 | 0 | 0 | 0 | 1 | 1 | 0 | 0 | 2 | Low |
| Bekele F et al., 2017 | 0 | 0 | 0 | 0 | 0 | 1 | 1 | 1 | 0 | 3 | Moderate |
| Gedefaw G et al., 2019 | 1 | 1 | 0 | 0 | 0 | 1 | 1 | 0 | 0 | 4 | Moderate |
| Bulto GA, 2019 | 0 | 0 | 0 | 0 | 0 | 0 | 0 | 0 | 0 | 0 | Low |
| Anchebi HT et al., 2017 | 0 | 0 | 0 | 0 | 0 | 1 | 1 | 0 | 0 | 2 | Low |
| Kedir T, 2017 | 0 | 0 | 0 | 0 | 0 | 1 | 1 | 0 | 0 | 2 | Low |
| Fisseha MA et al., 2017 | 0 | 0 | 0 | 0 | 0 | 1 | 1 | 0 | 0 | 2 | Low |
| Azage M et al., 2018 | 0 | 0 | 0 | 0 | 0 | 1 | 1 | 0 | 0 | 2 | Low |
| Gena HM, 2017 | 0 | 0 | 0 | 0 | 0 | 1 | 1 | 0 | 0 | 2 | Low |
| Abebe M, 2017 | 0 | 0 | 0 | 0 | 0 | 1 | 1 | 0 | 0 | 2 | Low |
| Niguse R et al., 2019 | 1 | 0 | 0 | 0 | 0 | 1 | 1 | 0 | 0 | 3 | Moderate |
| Shallo SA et al., 2018 | 0 | 0 | 0 | 0 | 0 | 1 | 1 | 0 | 0 | 2 | Low |
| Upashe SP et al., 2015 | 0 | 0 | 0 | 0 | 0 | 1 | 1 | 0 | 0 | 2 | Low |
| Gultie TK, 2014 | 0 | 0 | 0 | 0 | 0 | 1 | 1 | 0 | 0 | 2 | Low |
| Abera Y, 2004 | 0 | 0 | 0 | 0 | 0 | 1 | 1 | 0 | 0 | 2 | Low |
| Tegegne TK, 2014 | 0 | 0 | 0 | 0 | 0 | 1 | 1 | 1 | 0 | 3 | Moderate |
| Belayneh Z et al., 2019 | 0 | 0 | 0 | 0 | 0 | 1 | 1 | 0 | 0 | 2 | Low |
| Shumie ZS, 2021 | 0 | 0 | 0 | 0 | 0 | 1 | 1 | 0 | 0 | 2 | Low |
