# Supplementary material for: Menstrual hygiene practice among adolescent girls in Ethiopia: A systematic review and meta-analysis
Source: PLoS One. 2022 Jan 4;17(1):e0262295. doi: 10.1371/journal.pone.0262295 (PMC8726503; doi:10.1371/journal.pone.0262295)
Supplement: S4 File — (DOCX) [file pone.0262295.s004.docx]

**Supplementary file 3: Sensitivity analysis for estimates on safe MHM prevalence among adolescent girls in Ethiopia**

| **Author name** | Prevalence | 95%CI | I^2^ (%) | Heterogeneity chi-squared (Q) | p-value |
| --- | --- | --- | --- | --- | --- |
| Birhane AD et al., 2020 | 52.86 | 43.97-61.75 | 99.1 | 2313.43 | <0.001 |
| Felleke AA et al., 2021 | 52.55 | 43.70-61.39 | 99.1 | 2317.34 | <0.001 |
| Kitesa B et al., 2016 | 51.86 | 43.05-60.66 | 99.1 | 2261.01 | <0.001 |
| Zeleke B, 2016 | 51.17 | 43.08-59.27 | 98.9 | 1814.19 | <0.001 |
| Biruk E, 2017 | 52.70 | 43.71-61.69 | 99.1 | 2317.21 | <0.001 |
| Bekele F et al., 2017 | 52.03 | 43.22-60.83 | 99.1 | 2296.71 | <0.001 |
| Gedefaw G et al., 2019 | 52.87 | 44.00-61.74 | 99.1 | 2313.65 | <0.001 |
| Bulto GA, 2019 | 53.42 | 44.63-62.21 | 99.1 | 2269.71 | <0.001 |
| Anchebi HT et al., 2017 | 52.49 | 43.61-61.36 | 99.1 | 2316.17 | <0.001 |
| Kedir T, 2017 | 53.03 | 44.12-61.94 | 99.1 | 2300.26 | <0.001 |
| Fisseha MA et al., 2017 | 53.78 | 45.11-62.45 | 99.1 | 2196.50 | <0.001 |
| Azage M et al., 2018 | 54.05 | 45.95-62.14 | 98.9 | 1798.74 | <0.001 |
| Gena HM, 2017 | 52.42 | 43.46-61.39 | 99.1 | 2312.12 | <0.001 |
| Abebe M, 2017 | 53.05 | 44.02-62.08 | 99.1 | 2282.95 | <0.001 |
| Niguse R et al., 2019 | 51.81 | 43.03-60.58 | 99.1 | 2274.35 | <0.001 |
| Shallo SA et al., 2018 | 52.98 | 44.13-61.82 | 99.1 | 2310.71 | <0.001 |
| Upashe SP et al., 2015 | 53.30 | 44.42-62.19 | 99.1 | 2246.23 | <0.001 |
| Gultie TK, 2014 | 50.86 | 43.62-58.09 | 98.6 | 1425.37 | <0.001 |
| Abera Y, 2004 | 52.94 | 43.95-61.93 | 99.1 | 2303.96 | <0.001 |
| Tegegne TK, 2014 | 53.52 | 44.74-62.29 | 99.1 | 2247.56 | <0.001 |
| Belayneh Z et al., 2019 | 53.31 | 44.43-62.19 | 99.1 | 2247.53 | <0.001 |
| Shumie ZS, 2021 | 52.23 | 43.36-61.10 | 99.1 | 2303.68 | <0.001 |
